# Regulation (EC) No 1901/2006 on medicinal products for paediatric use & clinical research in vulnerable populations

**DOI:** 10.1186/1753-2000-2-37

**Published:** 2008-12-08

**Authors:** Birka Lehmann

**Affiliations:** 1 Bundesinstitut für Arzneimittel und Medizinprodukte, Kurt-Georg-Kiesinger Allee 3, 53175 Bonn, Germany

## Abstract

Before any medicinal product is authorised for use in adults, it must undergo extensive pharmaceutical consistency and stability tests, toxicological tests and clinical trials to ensure that it is of high quality, safe and effective.

The same approach may not always be applied to medicinal products used to treat children.

Studies showed that over 50% of the medicinal products used in children may not have been tested for use in this age group. The absence of suitable authorised medicinal products to treat conditions in children results from the fact that pharmaceutical companies do not adapt medicinal products to the needs of the paediatric population. This leaves health care professionals with no alternative other than to use medicinal products "off-label" and to use unauthorised products with the associated risks of inefficacy and/or adverse reactions.

The Regulation (EC) No 1901/2006 sets up a system of requirements, rewards and incentives, together with horizontal measures, to ensure that medicinal products are researched, developed and authorised to meet the therapeutic needs of children.

The Regulation is addressed to:

1. The pharmaceutical industry by setting out the legal framework for receiving rewards and incentives by conducting clinical trials in the paediatric population.

2. The Member States to set out to support research into, and the development and availability of, medicinal products for paediatric use.

3. The Community as funds for research into medicinal products for the paediatric population shall be provided for in the Community budget in order to support studies relating to medicinal products or active substances not covered by a patent or a supplementary protection certificate.

The legal framework for conducting clinical trials, including children/minors, is set up in Directive 2001/20/EC, the Clinical Trials Directive (CTD), for the European Union (EU). The CTD establishes specific provisions regarding conduct of clinical trials, including multi-centre trials, on human subjects involving medicinal products and in particular relating to the implementation of good clinical practice. Compliance with this good practice provides assurance that the rights, safety and well-being of trial subjects are protected, and that the results of the clinical trials are credible. The CTD is addressed to all investigators conducting clinical trials including clinical trials in the paediatric population and had to be applied accordingly.

In the framework of the authorisation of medicinal products regulated by the Regulation (EC) No 726/2004 and Directive 2001/83/EC as amended and the CTD, and additional implementing Directives and guidelines, the new Regulation (EC) No 1901/2006 is an important new piece of legislation focusing on the requirements to improve the situation for the paediatric population.

All Regulations/Directives to be found:

## Background

In contrast to the situation in adults, more than 50% of the medicines used to treat the children of Europe have not been adequately tested and are not authorised for use in children [1]. Therefore the health and thereof the quality of life of the children of Europe may suffer from a lack useful age appropriate medicinal products.

The paediatric population is not a homogeneous group it encompasses several subsets defined in ICH guideline E11: the pre term and term neonate from 0 to 27 days, the infant from 1 month to 23 months, the child from 2 years to 11 years and the adolescent from 12 up to 18 years [2].

Children are not miniature versions of adults. Due to age-related differences in drug handling or drug effects which may lead to different dose requirements to achieve efficacy or to avoid adverse reactions, specific clinical trials in paediatric populations are normally required. In addition, there may be practical problems of administration e.g. difficulties swallowing tablets if a syrup is not available or, more significantly, serious calculation errors when using adult formulations to obtain paediatric dosages. Children are a vulnerable group with developmental, physiological and psychological differences from adults, which makes age and development related research of medicines particularly important.

Although there may be concerns voiced about conducting trials in the paediatric population, this has to be balanced by the ethical issues related to giving medicines to a population in which they have not been tested and therefore their effects, positive or negative, are unknown. In order to address the concerns about trials in children it has to be pointed that the requirements for the protection of the paediatric population who take part in clinical trials in the Community laid down in Directive 2001/20/EC of the European Parliament and of the Council of 4^th ^April 2001.

In terms of both public health and ethics, it is clearly preferable to test medicines in children, in a safe and controlled clinical trial environment, where the individual child is protected and the studies generate data and information for the benefit of the rest of the children of the EU than to go on with the daily "experiments on children" that today occur because such medicines for children have never been designed and evaluated for this particular use [3].

In order to increase the availability of information on the use of medicinal products in the paediatric population, and to avoid unnecessary repetition of studies in the paediatric population European database provided for in Article 11 of Directive 2001/20/EC should include a European register of clinical trials of medicinal products for paediatric use – part of the information – should be made public by the EMEA.

The overall policy objective with the new Regulation is to improve the health of the children of Europe by increasing the research, development and authorisation of medicinal products for use in children.

General objectives are to:

• increase the development of medicinal products for use in children,

• ensure that medicinal products used to treat children are subject to high quality research,

• ensure that medicinal products used to treat children are appropriately authorised for use in children,

• improve the information available on the use of medicinal products in children.

• achieve these objectives without subjecting children to unnecessary clinical trials and in full compliance with the EU CTD.

To ensure that all the medicinal products required by children fall within the scope of the proposal and to fully understand the measures proposed, it is necessary to break medicinal products down into three groups:

- medicinal products in development (not yet to be authorised)

- authorised medicinal products still covered by patents or supplementary protection certificates

- authorised medicinal products not covered by these instruments.

The Regulation comprises several core elements in respect to collection of information on medicinal products, supported by a variety of rewards and incentives and penalties, the Paediatric Committee (PDCO) and transparency measures.

## 1. CORE element: data collection and verification

The Regulation reflects on three different actual situations for the collection of data for medicinal products in relation to the use in children.

The recommendation of the PDCO addressed to the MSs regarding the collection of data in the off-label use as required by Article 42 of the Regulation are published in the EMEAs' web-site. This is an on-going tasks for the MSs and no information is as today published.

Firstly the retrospective collection of information in accordance with Article 45 where it obliges the marketing authorisation holder of a medicinal product to provide all information regarding clinical trials in children which are already completed at latest by January 26 2008 to the competent authorities. The submitted data and the references to these data in the corresponding Package Leaflet (PL) and Summary of Product Characteristics (SmPC) will be evaluated in the framework of Paediatric Work-sharing Programme hosted by the coordination group (CMD) [4].

Secondly all on-going clinical trials have to be submitted within 6 months of their completion according to Article 46.

The third and far-reaching measures are laid down in Articles 7 and 8 of the Regulation.

Article 7 requires that by 26^th ^July 2008 all applications of new medicinal products only will be validated by the competent authorities with a Paediatric Investigation Plan (PIP) and results of studies according to this PIP or a PIP deferral of a PIP waiver.

The same applies for extensions of an already authorised medicinal product with a Supplementary Protection Certificate (SPC) or a Patent according to Article 8 from January 26^th ^2009 onwards.

An additional tool to improve the knowledge on medicinal products in the use of children is laid down in Article 30 the so-called Paediatric Use Marketing Authorisation (PUMA) where for off patent medicinal products data exclusivity are offered for authorised medicinal as an incentive.

All information which will be collected in the different routes of getting relevant recommendation to treat children with a medicinal product will be included in the PL and SmPC for each corresponding medicinal product in question.

## 2. CORE element: rewards, incentives & penalties, and sanctions

### 2.1 Rewards and Incentives (Articles 36 – 40)

The Regulation contains a shared responsibility between European Commission (EC) and Member States (MSs) in respect of incentives for research and development of medicinal products for paediatric use and for placing such products on the market, within the framework of their own powers and responsibilities.

The requirement for data in children applies to the current procedures for marketing authorisation applications; the reward for compliance with the requirement is an extension to the existing supplementary protection certificate; for orphan medicinal products (OMP) the reward for compliance with the requirement is two years added to the existing market exclusivity; the new type of marketing authorisation, the PUMA, utilises the current marketing authorisation procedures.

#### 2.1.1 EU

Patent protection, Supplementary Protection Certificate, market exclusivity, data exclusivity

For new medicinal products or line extensions of existing patented medicinal products, covered by a patent or a SPC, if all the measures included in the agreed paediatric investigation plan are complied with and if the product is authorised in all MSs and if relevant information on the results of studies is included in product information, the six-month SPC extension will be granted.

Because the reward is for conducting studies in children and not for demonstrating that a product is safe and effective in children, the reward will be granted even when a paediatric indication is not granted.

For OMPs a two-year extra market exclusivity will be rewarded.

Under the EU orphan drug Regulation, medicinal products designated as OMPs gain ten-years of market exclusivity on the granting of a marketing authorisation in the orphan indication. Therefore it is proposed to extend the ten-year period of orphan market exclusivity to twelve-years if the requirements for data on use in children are fully met.

The PUMA will utilise existing marketing authorisation procedures but is specifically for medicinal products developed exclusively for use in children. By allowing retention of the existing brand name and a benefit for the data protection time of 10 years associated with a new marketing authorisation will be rewarded.

#### 2.1.2 Member States

The rewards and incentives included in the Regulation do not preclude access of medicinal products being developed for children to other incentives or rewards by MSs. It is within their respective spheres of competence, to provide other incentives for developing medicinal products for paediatric use.

MSs are asked to provide information in this respect to the EC by a given time point and are asked to update the EC on a regularly basis.

### 2.2 Penalties and Sanctions (Article 49 – 50)

#### 2.2.1 EU

At the EMEA's request, the Commission may impose financial penalties for infringement of the provisions of this Regulation or the implementing measures adopted pursuant to it in relation to medicinal products authorised through the procedure laid down in Regulation (EC) No 726/2004. The maximum amounts as well as the conditions and methods for collection of these penalties shall be laid down in accordance with the procedure referred to in Article 51(2) of this Regulation.

The EC shall make public the names of anyone infringing the provisions of this Regulation or of any implementing measures adopted pursuant to it and the amounts of, and reasons for, the financial penalties imposed.

#### 2.2.2 Member States

At national level penalties for infringement of the Regulation

Without prejudice to the Protocol on the Privileges and Immunities of the European Communities, each MSs shall determine the penalties to be applied for infringement of the provisions of this Regulation or the implementing measures adopted pursuant to it in relation to medicinal products authorised through the procedures laid down in Directive 2001/83/EC and shall take all measures necessary for their implementation. The penalties shall be effective, proportionate and dissuasive.

The first inventory of Community and MSs rewards and incentives to support research into, and the development and availability of, medicinal products for paediatric use according to Article 39 of the Regulation, was made public on 30 July 2008 on the ECs web-site.

## 3. CORE element: implementation of the paediatric committee (PDCO)

Composition and tasks of the PDCO (Article 5–6)

### 3. 1. Composition

Regarding the composition of the new PDCO two aspects have to been taken into account by ensuring the continuity in the scientific and ethical considerations of the medicinal product in question.

The continuity of the scientific aspects is assured by the requirement that 5 member of the PDCO are also members of the Committee for Human Medicinal Product (CHMP) the opinion taking body in a marketing authorisation procedure for medicinal products. For the moment only the 4 members from Romania, Estonia, Lithuania and Slovak Republic are building the link. For the second aspect patients/parents and health professionals representatives are to be included in the PDCO. Each member has an alternate.

Information on the PDCO members and alternates are to be find on the EMEAs' web-site 

### 3.2 Tasks

Detailed information will be found in the references given in brackets after each bullet point.

The PDCO is asked to

• Assess and formulate opinions on PIPs, waivers and deferrals including consideration of whether proposed studies can be expected to be of significant therapeutic benefit and/or fulfil a therapeutic need of the paediatric population

• Advice on surveys regarding existing paediatric use [5].

• Support of the EMEA regarding the network of paediatric experts [6].

• Providing advice (on request) [7].

• Establishment of an inventory of paediatric needs [8].

The Regulation includes provisions for funding of studies into off-patent medicinal products. This funding, provided through the EU Framework programmes, should cover the development of off patent medicinal products with a view to the submission of a Paediatric Use Marketing

The objective of the priority list is to provide the basis for the work programme for the Third Call for Framework Programme 7 of the European Commission. It ensures that funds are directed into research of medicinal products with the highest need in the paediatric population.

The list of off-patent products has been revised by the PDCO and was agreed on 29/08/2008.

The list includes only products considered to be off-patent, i.e. not covered by a basic patent or a supplementary protection certificate. Information on the off-patent status is not guaranteed by EMEA. It should be noted that information on the authorisation status as well as on available paediatric formulations of medicinal products is very limited and not available for all European Member States. Users of this list are therefore advised to check the patent and authorisation status of the medicinal products of interest.

The methodology used to establish the list was based as much as possible on evidenced based medicine. It is however acknowledged that identification of priorities for research into medicinal products for paediatric use is partly based on subjective criteria and that identified priorities may change over time.

• Recommendation on a symbol

Article 32 of the Regulation (EC) No 1901/2006 foresees that medicinal products granted a marketing authorisation for a paediatric indication shall display a symbol for their identification. Following this Regulation, the selection of the symbol by the EC is to be based on a recommendation of the EMEAs' PDCO. The Regulation provides for the Commission to select the symbol by 26 January 2008 and make the symbol public. On the 20th of December 2007 the PDCO adopted its recommendation regarding the symbol by a majority vote of eighteen against four. The adopted recommendation is that

"As a consequence of its analysis balance of benefits and risks of the symbol, the Paediatric Committee was unable to recommend to the EC any symbol for which the benefits would outweigh the risks identified and dominated by potentially fatal medication errors".

Publication of this announcement serves to inform stakeholders that on the basis of this recommendation, the EC is at present not in a position to select a symbol and the provisions of Article 32 of the Regulation cannot therefore be implemented [3].

It is unclear for the moment in which way this provision shall be handled by the MSs as this also apply to medicinal products authorised before the entry into force of this Regulation.

### 3.3 Paediatric Investigation Plan (PIP)

The new key element of the Regulation is the early involvement of a company independent scientific and regulatory body, the PDCO, in the research and development programme of a medicinal product by the requirement to receive an agreement/a decision on the proposed process for a new medicinal product. Which contains two elements either to get a waiver or an agreement on the clinical trials, and if necessary including a deferral, in children to be included in the development programme.

The aim is to ensure that the necessary data are generated determining the conditions in which a medicinal product may be authorised to treat the paediatric population. The timing and the measures proposed to assess quality, safety and efficacy in all subsets of the paediatric population that may be concerned shall be presented in a PIP dossier. In addition, any measures to adapt the formulation of the medicinal product for its use in the paediatric population shall be included.

The content of information which shall be provided in a PIP is set out in the 'Commission guideline on the format and content of applications for agreement or modification of a paediatric investigation plan and requests for waivers or deferrals and concerning the operation of the compliance check and on criteria for assessing significant studies' [3].

## 4. Transparency and information

(Article 41 and 28)

One of the objectives of the Regulation is to increase the information available on the use of medicines for children. Through increased availability of information, the safe and effective use of medicinal products for children can be increased so promoting public health. In addition, availability of this information will help prevent the duplication of studies in children and the conduct of unnecessary studies in children. One of the measures is to build on the public health work of the CTD. The CTD establishes a Community database of clinical trials (EudraCT).

### 4.1 Transparency regarding clinical trials

Article 41 of the Regulation requires the Commission to draw up guidance on the nature of the information on paediatric clinical trials to be entered into the database of clinical trials (EudraCT), created by the CTD, on which information shall be made available to the public, on how clinical trials results shall be submitted and be made public and on the EMEAs' responsibilities and tasks in this regard.

The aim of the new Regulation is also to increase transparency in respect to clinical trials in children in all phases of the progress, beginning from the planning and recruiting of patients to the on-going and finalised studies.

This requirements goes much beyond the requests presented in the CTD where the access to the European database on clinical trials is limited to the competent authorities of the MSs, the EMEA and the EC and in Regulation (EC) No. 726/2004 Article 57 which is only reflecting on the publication of information on clinical trials for already authorised medicinal products.

The EC published a consultation on a "Draft Guidance on the information concerning paediatric clinical trials to be entered into the EU Database on Clinical Trials (EudraCT) and on the information to be made public by the EMEA, in accordance with Article 41 of Regulation No. (EC) 1901/2006".

### 4.2 Information

Article 28 of the Regulation sets out where authorisation is granted, the results of all those studies shall be included in the SmPC and, if appropriate, in the PL of the medicinal product, provided that the competent authority deems the information to be of use to patients, whether or not all the paediatric indications concerned were approved by the competent authority. Where a marketing authorisation is granted or varied, any waiver or deferral which has been granted pursuant to this Regulation shall be recorded in the SmPC and, if appropriate, in the PL of the medicinal product concerned. If the application complies with all the measures contained in the agreed completed PIP and if the summary of product characteristics reflects the results of studies conducted in compliance with that agreed PIP, the competent authority shall include within the marketing authorisation a statement indicating compliance of the application with the agreed completed PIP. For the purpose of the application of Article 45(3), this statement shall also indicate whether significant studies contained in the agreed PIP have been completed after the entry into force of this Regulation.

This has to be transposed by the revision of the guideline on the SmPC. In this respect to EC realised a public consultation in the beginning of 2008. The finalisation of the revision is still pending.

## 5. Supporting measures – guidelines

### 5.1. Guideline on Ethical consideration

To contribute to the protection of children who are the subject of clinical trials a specific recommendation was deemed to be necessary. Furthermore, the recommendations are intended to facilitate a harmonised application of rules on clinical trials across the EU and thereby facilitate the conduct of clinical trials in the EU.

Therefore the EC realised a guideline on 'Ethical considerations for clinical trials on medicinal products conducted with the paediatric population'.

Recommendations of the ad hoc group for the development of implementing guidelines for Directive 2001/20/EC relating to good clinical practice in the conduct of clinical trials on medicinal products for human use [3].

### 5.2 Guidelines on clinical trials

Additionally all guidelines containing recommendation on clinical trials for specific indication have to be carefully scrutinized and updated in respect to the requirements for conducting clinical trials in the paediatric population taking into account the different age groups [9].

## Conclusion

The Regulation (EC) No 1901/2006 sets up a system of requirements, rewards and incentives, together with horizontal measures, to ensure that medicinal products are researched, developed and authorised to meet the therapeutic needs of children.

The legal framework for conducting clinical trials, including children/minors, is set up in Directive 2001/20/EC for the European Union.

Harmonised ethical considerations are published by the European Commission to been taken into account by all interested parties conducting clinical trials in the paediatric population.

The Regulation includes provisions for funding of studies into off-patent medicinal products. This funding, provided through the EU Framework programmes, should cover the development of off patent medicinal products with a view to the submission of a Paediatric Use Marketing

It is now of utmost importance to set the scene in the European Union to convince the paediatric patients, parents, caretaker, nurses and doctors to assent and consent in participating in clinical trials for the benefit of the paediatric population by large.

## Abbreviations

CMDh: Coordination group human according to Article 27 Directive 2001/83/EC; CTD: Clinical Trial Application; EC: European Commission; EMEA: European Medicines Agency; EU: European Union; EudraCT: European clinical trials database; MS(s): Member State(s); OMP: Orphan Medicinal Product; PDCO: Paediatric Committee; PIP: Paediatric Investigation Plan; PL: Package Leaflet; PUMA: Paediatric Use marketing Authorisation; SmPC: Summary of Product Characteristics; SPC: Supplementary Protection Certificate.

## Competing interests

The authors declare that they have no competing interests.
